# Q-Needle-Assisted Intraductal Injection Enhances Dacryoendoscopic Surgery for Primary Acquired Lacrimal Drainage Obstruction: A Retrospective Study

**DOI:** 10.3390/jcm15082954

**Published:** 2026-04-13

**Authors:** Doah Kim, Siyun Lee, Helen Lew

**Affiliations:** Department of Ophthalmology, CHA Bundang Medical Center, CHA University, Seongnam 13496, Gyeonggi-do, Republic of Korea; inhamed93216006@gmail.com (D.K.);

**Keywords:** anti-inflammatory agents, dacryoendoscopy, dexamethasone, drug delivery systems, epiphora, fluorouracil, lacrimal duct obstruction, triamcinolone

## Abstract

**Background/Objectives**: Primary acquired lacrimal drainage obstruction (PALDO) is a common cause of epiphora. Although dacryoendoscopic recanalization (DER) is widely performed, its long-term success is limited by restenosis related to fibro-inflammatory processes. This study aimed to evaluate the efficacy of a novel Q-needle for targeted intraductal delivery of antifibrotic and anti-inflammatory agents during DER. **Methods**: A retrospective review was performed on 190 eyes treated with DER, silicone tube intubation (SI), and retrograde intraductal injection via the inferior meatus using a Q-needle. A mixture of dexamethasone (1 mL), 5-fluorouracil (1 mL), and triamcinolone acetonide (1 mL) was administered directly into the obstruction site under endoscopic visualization. Obstruction type was classified intraoperatively as secretory or structural based on dacryoendoscopic findings. **Results**: The overall surgical success rate was 92.1%, with significantly greater success in secretory-type PALDO compared to the structural type (96.8% vs. 87.4%, *p* = 0.031). These outcomes contrast with previous reports in which secretory-type PALDO was associated with poorer prognosis after DER. **Conclusions**: The improved outcomes in the secretory group suggest a potential role of combined antiproliferative and multi-phase anti-inflammatory therapy in effectively addressing the key mechanisms of restenosis. Q-needle–assisted intraductal injection during DER may represent a simple and safe adjunctive approach to improve surgical consistency and long-term patency in patients with PALDO.

## 1. Introduction

Primary acquired lacrimal drainage obstruction (PALDO) is a common cause of epiphora, recurrent dacryocystitis, and ocular discomfort, significantly impairing patients’ quality of life [1]. The advent of dacryoendoscopy has enabled direct visualization of the lacrimal drainage system, enhancing diagnostic accuracy and facilitating targeted treatment [2]. Dacryoendoscopic recanalization (DER) combined with silicone tube intubation (SI) is widely used as a minimally invasive surgical option; however, its success rate remains variable depending on the underlying pathology [3].

Our previous work demonstrated an overall success rate of 86.2% for DER with SI, with notably higher success in structural-type PALDO (95.3%) than in secretory-type PALDO (79.7%) [3,4]. This disparity underscores the need for an effective strategy to improve outcomes in secretory-type PALDO, where inflammation, mucus accumulation, and mucosal dysfunction play a larger role in pathogenesis.

Surgical failure in PALDO is often related to postoperative mucosal scarring and fibrosis at the drainage site. Corticosteroids and antiproliferative agents such as mitomycin C (MMC) have been shown to modulate the inflammatory and proliferative phases of wound healing, respectively [5]. In dacryocystorhinostomy (DCR), the combination of MMC with triamcinolone acetonide (TA) has achieved anatomical success rates exceeding 90% [5,6], and both topical and intramucosal delivery methods have been explored to enhance postoperative outcomes [7,8]. However, a standardized approach for targeted intraductal drug delivery during DER has not been established.

The question mark-shaped Q-needle enables retrograde intraductal administration of therapeutic agents directly to the nasolacrimal duct (NLD) under endoscopic visualization. This technique allows simultaneous confirmation of ostium patency and precise delivery of medications to sites of active inflammation and fibrosis. In this study, we evaluated the clinical efficacy of Q-needle-assisted intraductal injection of dexamethasone, 5-fluorouracil (5-FU), and TA during DER, with the aim of improving surgical success rates—particularly in secretory-type PALDO—and identifying factors associated with treatment outcomes.

## 2. Materials and Methods

### 2.1. Study Design and Subjects

This study aimed to evaluate the efficacy of Q-needle-assisted intraductal drug delivery as an adjunct to dacryoendoscopic recanalization (DER). It was designed as a retrospective, single-center study conducted at Bundang Medical Center, CHA University, between March 2022 and January 2024. A total of 170 patients (190 eyes) with PALDO were included. Cases with PALDO secondary to systemic inflammatory disorders, and neoplasia—including primary lacrimal system tumors, secondary invasion from adjacent tissues, or even distant metastasis—were excluded. Clinical histories, duration of epiphora, tear meniscus height (TMH), canaliculus irrigation tests, and dacryocystography (DCG) were examined.

DCG was performed to evaluate the obstructed site, using a water-soluble contrast agent, iohexol (Bonorex^®^; Central Medical Service, Seoul, Republic of Korea). DCG findings were interpreted as follows: Primary obstruction primarily involves structural changes within the NLD itself, ranging from simple narrowing to complete blockage, hindering the normal flow of tears. Conversely, secondary obstruction is characterized by features such as beading along the duct or dilation of the lacrimal sac [3].

The type of obstruction was defined according to dacryoendoscopic findings within the occluded segment, based on video images acquired from the canaliculus to the inferior meatus. Obstructions were classified into two groups: secretory and structural. Secretory obstruction was defined by predominant intraluminal mucoid material and secretion-associated lesions (e.g., dacryoliths and granulation tissue), whereas structural obstruction was defined by fixed narrowing such as fibrotic membranes or stenosis without predominant intraluminal secretions [3,4]. All classifications were performed by a single experienced surgeon (H.L.) to ensure consistency. Patients with granulation tissue and/or dacryoliths were not excluded; these findings were handled within the recanalization workflow, and Q-needle injection was used as adjunctive therapy rather than as a stand-alone treatment.

### 2.2. Surgical Procedure

All surgeries were performed under local anesthesia with topical lidocaine. The overall technique was based on a previously reported dacryoendoscopy-guided silicone intubation procedure [4], modified to incorporate Q-needle-assisted intraductal injection as an adjunct after mechanical recanalization.

A dacryoendoscope (FT-203F; Fibertech, Tokyo, Japan) and a 2.7 mm nasal endoscope (7208CA; Karl Storz, Tuttlingen, Germany) were used to visualize and treat the lacrimal drainage system (LDS). The dacryoendoscope provided magnified visualization of the intraluminal structure, allowing precise identification of the stenotic segment. A flexible sheath was mounted on the scope to enable gentle dilation and advancement within the ductal lumen under continuous visual control.

Once the obstruction site was localized, the blocked segment was mechanically recanalized by advancing and withdrawing the sheath as a micro-dilator to restore the continuity of the nasolacrimal passage. Dacryoendoscopic intraluminal lesions encountered during recanalization (including granulation tissue and/or dacryoliths) were managed within the recanalization step as part of restoring patency under direct visualization. Patency of the inferior meatus opening was then verified endonasally to confirm successful recanalization.

**Figure 1:**
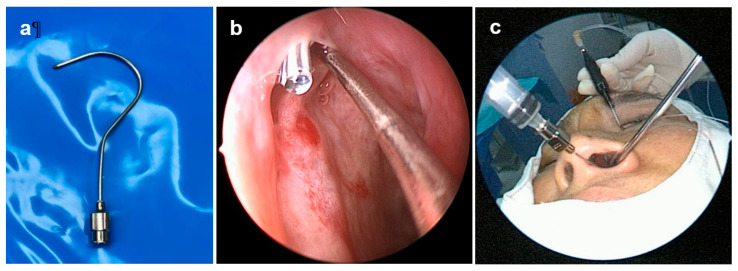
Photographs demonstrating the use of the Q-needle for retrograde irrigation during silicone tube intubation. (**a**) The Q-needle features a fine, curved design that allows direct delivery of irrigation medication into the nasolacrimal duct. (**b**) An intraoperative nasoendoscopic image shows the Q-needle positioned at the inferior meatus, indicating the entry point for retrograde irrigation and the exact location where the fluid was delivered. (**c**) An external surgical field view showing the simultaneous use of a nasal endoscope, a dacryoendoscope, and the Q-needle during retrograde irrigation. The curved tip of the Q-needle is inserted through the inferior meatus under endoscopic guidance, while the dacryoendoscope is introduced through the upper punctum to visualize the nasolacrimal duct lumen.

After the lumen was reopened, a Q-needle (Figure 1a) was inserted retrogradely from the inferior meatus toward the lacrimal sac for targeted intraductal drug delivery. A total of 3 mL of therapeutic mixture—1 mL each of 5-FU (250 mg/5 mL; JW Pharmaceutical, Seoul, Republic of Korea), TA (40 mg/mL; Dong Kwang, Seoul, Republic of Korea), and dexamethasone (5 mg/mL; Yuhan, Seoul, Republic of Korea)—was slowly injected to provide combined antifibrotic and anti-inflammatory effects along the treated duct. The step-by-step workflow of Q-needle placement, position confirmation, and intraductal injection is provided as Supplementary Video S1.

Finally, a bicanalicular silicone tube (0.94 mm; Yoowon Meditec, Seoul, Republic of Korea) was inserted under endoscopic guidance, and its ends were secured beneath the inferior turbinate. Postoperative management included topical moxifloxacin 0.5% (Vigamox; Alcon, TX, USA) and fluorometholone 0.1% (Flumetholon; Santen, Osaka, Japan), applied three times daily. Silicone tube removal was planned to occur at approximately six months, with timing individualized according to clinical findings. All surgeries were performed by a single experienced oculoplastic surgeon (H.L.).

### 2.3. Q-Needle Placement and Drug Delivery

The Q-needle was introduced through the nasal cavity and advanced along the lateral nasal wall. It was then carefully redirected superiorly toward the opening of the inferior meatus (Figure 1b). This technique allowed placement within the distal nasolacrimal duct under direct nasal endoscopic visualization before administering the 5-FU, TA, and dexamethasone mixture. The injection was delivered under direct nasal endoscopic visualization, ensuring uniform distribution of the agents along the treated segment. The overall intraoperative setup, including simultaneous use of the dacryoendoscope, nasal endoscope, and Q-needle-assisted intraductal injection, is demonstrated (Figure 1c).

### 2.4. Outcome Measures

Clinical characteristics, dacryoendoscopic findings, and surgical outcomes were evaluated based on medical records. Surgical success was defined as a Munk score of 0–1, a TMH of <300 μm, and a positive irrigation test confirming NLD patency. For the irrigation test, the “passed” category included both complete passage and partial passage (i.e., partial patency with some resistance and/or partial reflux), whereas “no passage” indicated no patency.

Postoperative follow-up visits were scheduled at 1 week, 1 month, 3 months, and approximately 6 months after surgery. Silicone tube extubation was planned at approximately 6 months and individualized based on clinical findings, followed by a visit 1 month after tube removal. At each visit, the Munk score, TMH measurement, and saline irrigation test were recorded.

### 2.5. Statistical Analysis

All statistical analyses were performed using SPSS software (version 26.0, IBM Corp., Armonk, NY, USA). Continuous variables were presented as mean ± standard deviation (SD) or median (interquartile range, IQR), depending on data distribution, and categorical variables as counts and percentages (%). Group comparisons were performed using the independent *t*-test or Mann–Whitney U test for continuous variables and the chi-square test or Fisher’s exact test for categorical variables. A *p*-value < 0.05 was considered statistically significant. Time-to-failure outcomes were analyzed using Kaplan–Meier survival curves and compared using the log-rank test. To address non-independence in bilateral cases, one eye per patient was randomly selected for Kaplan–Meier and Cox proportional hazards analyses. Multivariable Cox proportional hazards regression was performed, including age, sex, level of obstruction, and type of obstruction, and hazard ratios with 95% confidence intervals were reported.

### 2.6. Ethical Approval

This study was approved by the Institutional Review Board of Bundang Medical Center, CHA University (Approval No. 2024-07-049 and date of approval 2 September 2024). The requirement for informed consent was waived due to the retrospective nature of the study.

## 3. Results

### 3.1. Patient Demographics

A total of 170 patients (190 eyes) with PALDO were included in the study. Among these, 20 patients had bilateral surgery. The mean age of the patients was 60.4 ± 21.3 years, with a higher proportion of female patients (67.6% female, 32.4% male). Before treatment, the mean Munk score was 3.6 ± 1.0, indicating significant epiphora, and the mean TMH was 390.6 ± 268.7 μm. The irrigation test revealed complete obstruction in 42.1% of cases, while 57.9% exhibited partial patency. The mean silicone tube retention period was 5.9 ± 0.9 months, and the mean postoperative follow-up duration (from surgery to the last clinic visit) was 6.6 ± 1.0 months. Surgical success was assessed according to the predefined follow-up schedule, including a visit 1 month after silicone tube removal. Dacryoendoscopic examination revealed an equal distribution between structural-type and secretory-type PALDO, with each accounting for 50.0% (95 eyes each). The overall surgical success rate was 92.1% (175/190 eyes) (Table 1).

### 3.2. Association of Clinical Variables with Surgical Success

Among the 190 eyes that underwent DER and SI using the Q-needle, neither previous lacrimal surgical history (*p* = 0.075) nor irrigation test results (*p* = 0.083) showed a statistically significant association with surgical success. A statistically significant difference in success rates was observed based on dacryoendoscopic findings (*p* = 0.031). The success rate was significantly higher in the secretory type (96.8%) compared to the structural type (87.4%) (Table 2).

### 3.3. Surgical Success Rates by Obstruction Site

**Figure 2:**
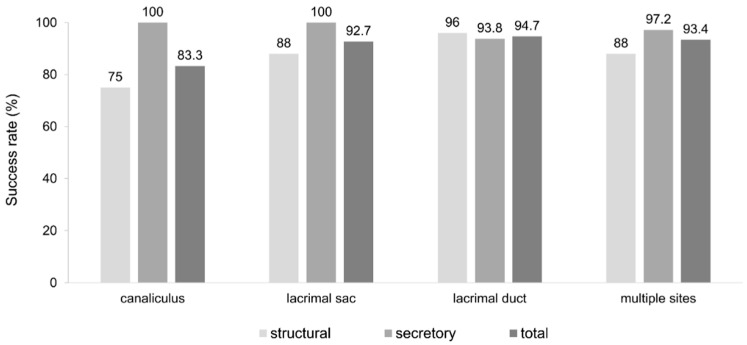
Surgical success rates stratified by the site of nasolacrimal duct obstruction, as identified on dacryoendoscopy. All patients underwent retrograde nasolacrimal duct irrigation using the Q-needle, followed by silicone tube intubation. Success rates were 83.3% for canalicular obstruction, 92.1% for lacrimal sac obstruction, 95.6% for lacrimal duct obstruction, and 90.6% for multiple sites (defined as obstruction at two or more sites).

The surgical success rate was analyzed according to the anatomical location of obstruction identified on dacryoendoscopy. Patients were categorized into four subgroups according to the obstruction site: canaliculus, lacrimal sac, lacrimal duct, and multiple sites (defined as obstruction at two or more locations). The lacrimal duct group showed the highest success rate (94.7%), followed by multiple sites (93.4%), lacrimal sac (92.7%), and canaliculus (83.3%). Although canalicular obstruction showed a relatively lower success rate, the differences among the groups were not statistically significant (*p* = 0.174). These results are summarized in Figure 2.

### 3.4. Cumulative Success Rate over Time

**Figure 3:**
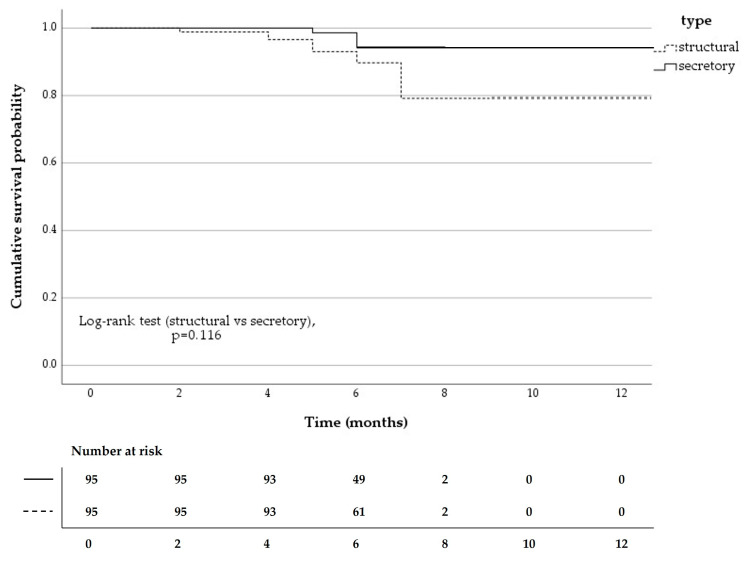
Kaplan–Meier survival curves showing the cumulative surgical success rate in patients who underwent dacryoendoscopic recanalization and silicone tube intubation with retrograde nasolacrimal duct injection using the Q-needle. Surgical success was defined as a Munk score of 0–1, TMH < 300 µm, and a positive irrigation test result. The curves represent the structural (dashed line) and secretory (solid line) types of obstruction. Log-rank test, *p* = 0.116.

Long-term surgical outcomes were assessed using Kaplan–Meier survival analysis in an independent-eye dataset (one eye per patient; *n* = 180), with one eye randomly selected in bilateral cases. The cumulative success rate gradually declined, reaching 90.5% by 12 months postoperatively. Most surgical failures occurred between 4 and 7 months and were more frequent in the structural group, whereas failures were rare in the secretory group. The survival curves did not differ significantly between the two groups (log-rank test, *p* = 0.116) (Figure 3). No intraoperative or postoperative adverse events were observed.

### 3.5. Multivariable Cox Proportional Hazards Regression

In multivariable Cox proportional hazards regression analysis adjusting for age, sex, and level of obstruction, secretory obstruction (vs structural obstruction) showed a trend toward lower failure risk in the secretory group compared with the structural group (HR, 0.28; 95% CI, 0.077–1.015; *p* = 0.053) (Table 3; Supplementary Figure S1).

## 4. Discussion

Dacryoendoscopy has emerged as a valuable technique for the direct visualization of the lacrimal drainage system, enabling precise identification of obstruction types such as stenosis, mucoceles, and dacryoliths [9,10,11]. Previous studies have demonstrated that modified transcanalicular endoscopic recanalization, guided by anatomical principles, can achieve high success rates (up to 95.6%) even in complete PALDO with longstanding fibrotic changes [12]. Tamao et al. reported that 88.7% of 124 lacrimal passage obstructions retained patency using dacryoendoscopic recanalization. They also assessed the outcomes based on the patients’ symptom scores, such as tearing, ocular discharges, swelling, pain, irritation, and blurred vision. Tearing was a dominant symptom in the pre-sac obstruction group, while others were frequent in the post-sac obstruction group [13].

The success rates following dacryoendoscopic treatment with Nunchaku-type silicone tube intubation at 12 months post-surgery showed that it was more successful for upper obstruction (94.6%) than for lower obstruction (71.4%) [14]. In addition, the female patients tended to have a successful outcome at 6 months post-lacrimal surgery, and then the tendency was reduced at 12 months post-surgery. The diameter of the lower lacrimal duct and the delayed mucosal healing speed have been suggested as the reasons for the sex-related differences in the incidence of lacrimal duct obstruction [14]. They also found that factors associated with a higher risk of failure of dacryoendoscopy-assisted laser dacryoplasty with silicone intubation reoperation include a longer duration of epiphora [15] and a history of chronic dacryocystitis [14,15]. However, we could not find the relationship between the success rate and the other factors.

Most of the studies regarding dacryoendoscopic recanalization focused on the Asian population (83.3%) and had a high female-to-male ratio (81.3%) [16]. Regarding various clinical factors, ethnic and sex variations in the anatomical structures of LDS [17] may influence the diagnostic and therapeutic success of dacryoendoscopic-guided recanalization. Furthermore, the 5-FU duration of the studies ranged from 2 weeks [18] to 25 months in congenital nasolacrimal duct obstruction [19], which may not be long enough to establish long-term significance. It should be considered that long-term outcomes and safety issues may be underreported or inconclusive, although the studies reported high therapeutic success and low complication rates [16]. The success rate of direct silicone intubation for nasolacrimal duct obstruction is approximately 52.5% at 8 to 30 months after tube removal and 62.5% at 3 months, indicating a high risk of recurrence in the long-term postoperative period. Factors associated with recurrence include a history of dacryocystitis and long disease duration [20].

In structural obstructions, particularly post-sac PALDO, success rates of dacryoendoscopic recanalization (DER) are significantly higher than in secretory or functional PALDO, where inflammation, mucus overproduction, and mucosal dysfunction play a greater role in pathogenesis [3]. Our prior cytological analyses demonstrated that mucinous obstruction is associated with a higher prevalence of bacterial colonization and neutrophilic infiltration than membranous obstruction, suggesting an ascending inflammatory process from the NLD to the lacrimal sac [21]. This inflammatory pathway, possibly mediated by mucosal sodium–iodide symporter disruption [22], may underlie delayed tear drainage and treatment resistance in functional PALDO.

Kaplan–Meier survival analysis further highlighted the temporal pattern of surgical outcomes. Most failures occurred between 4 and 7 months postoperatively, after which the cumulative success rate stabilized. After accounting for non-independence in bilateral cases (one eye per patient; *n* = 180), the survival curves showed a trend toward higher survival in the secretory group but did not reach statistical significance (log-rank test, *p* = 0.116). Although a higher success rate was observed in the secretory type in Table 2 (*p* = 0.031), this difference was not statistically significant in the Kaplan–Meier analysis and was only borderline in the Cox regression model. Therefore, this finding should be interpreted with caution and may suggest a potential benefit of Q-needle-assisted intraductal drug delivery in secretory-type obstruction, which may require further investigation. These findings suggest that the postoperative course is largely determined within the first several months, particularly between 4 and 7 months, and that secretion-related obstructions may respond more favorably to Q-needle-guided intraductal therapy. However, given the limited sample size, these findings should be interpreted with caution, and their generalizability remains restricted. The latter portion of the Kaplan–Meier curve should be interpreted with caution because fewer cases remained at risk at longer follow-up times. The relatively short follow-up period may limit the assessment of long-term surgical outcomes.

In our previous study using DER without intraductal drug delivery, the structural type showed a significantly higher success rate than the secretory type [3]. In the present study, however, a higher success rate was observed in the secretory type after Q-needle-assisted intraductal drug delivery. This difference may reflect a potential benefit of intraductal drug delivery in secretory-type PALDO, which has shown relatively lower success rates in prior reports. However, direct comparison between studies is limited, and the absence of a control group should be considered when interpreting these findings. Further studies, including a control group, are needed to clarify the role of intraductal drug delivery.

Given the multifactorial nature of secretion-related PALDO, adjuvant intraductal drug delivery represents a promising strategy to target both fibrosis and inflammation. The Q-needle, introduced retrograde through the valve of Hasner under nasal endoscopic guidance, allows precise intraductal administration of therapeutic agents while simultaneously confirming ostium patency. This approach allows direct delivery of medications to sites of pathological change, addressing inflammation, fibroblast proliferation, and mucus accumulation—three key contributors to surgical failure. The injected drug mixture was delivered via retrograde intraductal injection under direct nasal endoscopic visualization after recanalization, although the exact uniformity and depth of tissue distribution, particularly in partially or irregularly obstructed ducts, could not be directly assessed. Although postoperative dacryoendoscopic evaluation was not performed to directly assess mucosal irritation, no clinically significant adverse events were observed, and previous studies have suggested acceptable local safety of 5-FU in lacrimal surgery [23,24,25,26,27].

Antiproliferative agents such as MMC have been shown to reduce granulation tissue and maintain ostium patency after lacrimal bypass procedures; however, their strong, non-cell-cycle-specific DNA crosslinking effect increases the risk of late tissue thinning and necrosis [6,8,28,29,30]. In contrast, 5-FU, a pyrimidine analog that inhibits thymidylate synthase during the S-phase, provides a milder, more controllable suppression of fibroblast proliferation with a favorable safety profile and the flexibility for postoperative titration [31]. In lacrimal surgery, adjuvant 5-FU was applied to reduce restenosis risk while preserving mucosal integrity, particularly in inflammatory or secretion-predominant cases [32].

In parallel, intraductal administration of prednisolone acetate ointment has been reported as a successful approach for managing granulation tissue formation after SI [33]. Corticosteroids offer complementary anti-inflammatory benefits. TA, with its low solubility, provides long-lasting local suppression of inflammation, and a single local injection is generally considered to exert clinically relevant effects for several weeks [34,35]. Dexamethasone, in contrast, is water-soluble, with a rapid onset and a shorter duration of action, typically acting over several days and thereby targeting the acute postoperative inflammatory surge [35]. Brief intraoperative exposure to 5-FU has been associated with reduced scarring in glaucoma filtering and lacrimal bypass surgery [31,32,36]. The combination of 5-FU with both TA and dexamethasone, therefore, enables a staged anti-inflammatory and antifibrotic effect—dexamethasone addressing the immediate inflammatory response, TA maintaining prolonged suppression, and 5-FU controlling fibroproliferation. This triple-drug intraductal regimen, delivered precisely via the Q-needle, targets the key mechanisms of surgical failure in secretory PALDO: inflammation, fibrosis, and mucus overproduction. Accordingly, its aim is not to maintain continuous drug bathing of the NLD for months, but rather to achieve high local tissue concentrations during the critical early days to weeks of mucosal wound healing, when the fibro-inflammatory cascade that ultimately leads to restenosis is largely determined. We acknowledge that, once patency is restored and silicone intubation is in place, a portion of the injected solution may drain into the nasal cavity relatively quickly, and postoperative lacrimal irrigation during follow-up may further reduce residual intraluminal drug. Therefore, the effective local exposure time is likely variable across patients depending on postoperative patency and irrigation patterns.

Importantly, careful endoscopic guidance during recanalization is essential to avoid false passage formation, which can cause additional mucosal injury and compromise long-term outcomes [30,37]. Our modified DER technique, incorporating Q-needle-guided triple-drug delivery, proved particularly beneficial in patients with prior failed lacrimal surgeries, offering a minimally invasive yet mechanistically comprehensive intervention.

This study has several limitations, including its retrospective single-center design, relatively small sample size, limited follow-up duration, and lack of a randomized control group. We evaluated only a single intraoperative intraductal injection via the Q-needle (without repeated dosing), and several commonly recognized prognostic variables (e.g., duration of symptoms, revision status, and history of dacryocystitis) could not be reliably incorporated due to incomplete or unstandardized documentation. Moreover, intraluminal washout after recanalization cannot be standardized, and the qualitative dacryoendoscopic classification may not be universally standardized. In addition, as all surgeries were performed by a single experienced surgeon, the outcomes may partly reflect operator expertise, which may limit generalizability.

## 5. Conclusions

Meticulous dacryoendoscopic recanalization combined with targeted intraductal administration of 5-FU, TA, and dexamethasone via the Q-needle may represent a safe and pathophysiology-based adjunctive strategy that may help improve surgical outcomes in secretory PALDO and postoperative patency by addressing early fibro-inflammatory wound-healing responses while preserving mucosal health.

## Figures and Tables

**Table 1:** Demographic and clinical characteristics of patients with acquired nasolacrimal duct obstruction who underwent combined nasolacrimal duct irrigation using a Q-needle during dacryoendoscopic recanalization and silicone tube intubation.

Patients (Eyes)	170 (190)
Age (year)	60.4 ± 21.3
Male:female (%)	55:115 (32.4:67.6)
Munk score	3.6 ± 1.0
Tear meniscus height (μm)	390.6 ± 268.7
Irrigation test, pass:not pass (%)	110:80 (57.9:42.1)
Duration of the insertion (months)	5.9 ± 0.9
Follow-up period (months)	6.6 ± 1.0
Dacryoendoscopic findings	
Structural type:secretory type (%)	95:95 (50.0:50.0)
Success rate, % (number of eyes)	92.1 (175)

**Table 2:** Clinical outcomes of dacryoplasty and silicone tube intubation using retrograde nasolacrimal duct irrigation via the inferior meatus with the Q-needle in patients with acquired nasolacrimal duct obstruction.

	Success (%) (*n* = 175)	Failure (%) (*n* = 15)	Total (*n* = 190)	*p*-Value
History of allergy				
Yes	39 (97.5)	1 (2.5)	40	0.274
No	136 (90.7)	14 (9.3)	150	
Previous lacrimal surgery *				
Yes	35 (94.6)	2 (5.4)	37	0.775
No	140 (91.5)	13 (8.5)	153	
Lacrimal irrigation test				
Passed	105 (95.5)	5 (4.5)	110	0.083
Not passed	70 (87.5)	10 (12.5)	80	
Dacryocystographic findings				
Primary pattern	116 (92.8)	9 (7.2)	125	0.835
Secondary pattern	59 (90.8)	6 (9.2)	65	
Dacryoendoscopic findings				
Secretory type	92 (96.8)	3 (3.2)	95	0.031 **
Structural type	83 (87.4)	12 (12.6)	95	

* Previous lacrimal surgery includes balloon dacryoplasty and silicone tube intubation. ** *p*-value < 0.05. Statistical analysis was performed using the chi-square test.

**Table 3:** Multivariable Cox proportional hazards regression for time to surgical failure after dacryoendoscopic recanalization with Q-needle-assisted intraductal injection.

Variable	*p*-Value	Hazard Ratio	95% CI
Age	0.676	1.008	0.970–1.048
Sex (Male vs. Female)	0.577	0.720	0.228–2.279
Obstruction site *	0.624	0.742	0.224–2.452
Obstruction type (secretory vs. structural)	0.053	0.280	0.077–1.015

* Obstruction site: single site (canaliculus, lacrimal sac, or nasolacrimal duct) vs. combined (≥2 sites).

## Data Availability

The datasets generated and/or analyzed during the current study are not publicly available due to patient privacy and ethical restrictions, but are available from the corresponding author on reasonable request with permission of the Institutional Review Board.

## Supplementary Materials

The following supporting information can be downloaded at https://www.mdpi.com/article/10.3390/jcm15082954/s1, Figure S1: Adjusted hazard ratio for surgical failure comparing secretory and structural obstruction types; Video S1: Step-by-step Q-needle placement, position confirmation, and intraductal injection during dacryoendoscopic recanalization.

## Abbreviations

The following abbreviations are used in this manuscript:

5-FU	5-fluorouracil
CI	Confidence interval
DCG	Dacryocystography
DCR	Dacryocystorhinostomy
DER	Dacryoendoscopic recanalization
IQR	Interquartile range
LDS	Lacrimal drainage system
MMC	Mitomycin C
NLD	Nasolacrimal duct
PALDO	Primary acquired lacrimal drainage obstruction
SD	Standard deviation
SI	Silicone tube intubation
TA	Triamcinolone acetonide
TMH	Tear meniscus height
