# Supplementary figures and images for: Q-Needle-Assisted Intraductal Injection Enhances Dacryoendoscopic Surgery for Primary Acquired Lacrimal Drainage Obstruction: A Retrospective Study
Source: J Clin Med. 2026 Apr 13;15(8):2954. doi: 10.3390/jcm15082954 (PMC13115716; doi:10.3390/jcm15082954)

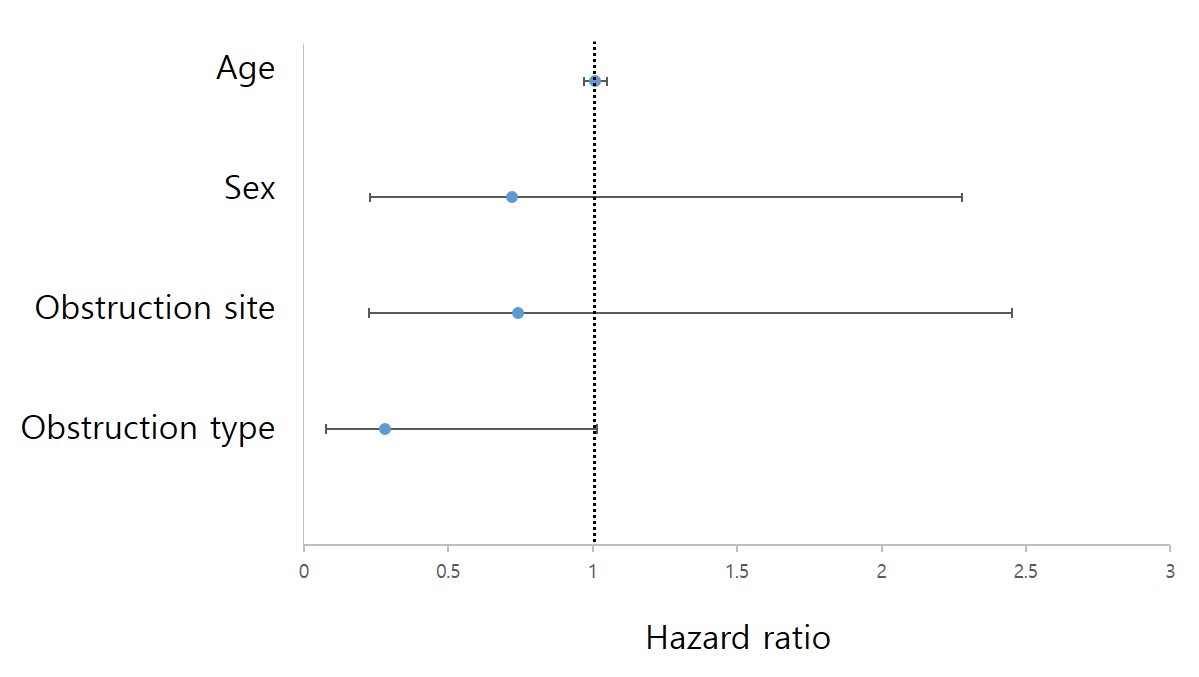

Supplement: Supplementary file 1 [file jcm-15-02954-s001.zip › Supplementary Figure S1.jpg]
